# Supplementary material for: Interpretable detection of epiretinal membrane from optical coherence tomography with deep neural networks
Source: Sci Rep. 2024 Apr 11;14:8484. doi: 10.1038/s41598-024-57798-1 (PMC11009346; doi:10.1038/s41598-024-57798-1)
Supplement: Supplementary file 1 — Supplementary Information. [file 41598_2024_57798_MOESM1_ESM.pdf]

Supplementary Data

Interpretable Detection of Epiretinal Membrane from Optical Coherence Tomography with Deep Neural Networks

Murat Seçkin Ayhan[1,§], Jonas Neubauer[2,§], Mehmet Murat Uzel[2,3], Faik Gelisken[2,‡], and Philipp Berens[1,4,*]

A.1 Classification performance

Supplementary Table 1: ERM detection accuracy of individual networks and their ensembles for various degrees of mixing (indicated by α) and corresponding numbers of epochs as given in Section 2.2.1. Gray rows indicate the models of choice based on validation accuracy.

			ResNet50			*InceptionV3*		
			Training	Validation	Test	Training	Validation	Test
Single	$\alpha = 0.0$		0.9803	0.9625	0.9435	0.9734	0.9634	0.9358
	$\alpha = 0.1$		0.9686	0.9625	0.9352	0.9746	0.9607	0.9282
	$\alpha = 0.2$		0.9506	0.9625	0.9371	0.9630	0.9616	0.9346
	$\alpha = 0.3$		0.9734	0.9634	0.9511	0.9643	0.9607	0.9447
	$\alpha = 0.4$		0.9790	0.9651	0.9441	0.9731	0.9651	0.9454
Ensemble	$\alpha = 0.0$	DNN 1	0.9722	0.9634	0.9454	0.9785	0.9607	0.9352
		DNN 2	0.9813	0.9642	0.9257	0.9398	0.9607	0.9136
		DNN 3	0.9640	0.9651	0.9333	0.9776	0.9607	0.9358
		DNN 4	0.9745	0.9660	0.9479	0.9856	0.9634	0.9390
		DNN 5	0.9897	0.9651	0.9428	0.9727	0.9564	0.9422
		Ensemble	0.9861	0.9686	0.9492	0.9843	0.9686	0.9473
	$\alpha = 0.1$	DNN 1	0.9631	0.9625	0.9543	0.9634	0.9616	0.9441
		DNN 2	0.9688	0.9703	0.9390	0.9827	0.9747	0.9365
		DNN 3	0.9729	0.96422	0.9358	0.9489	0.9668	0.9314
		DNN 4	0.9710	0.9651	0.9473	0.9807	0.9599	0.9365
		DNN 5	0.9644	0.9607	0.9441	0.9676	0.9651	0.9460
		Ensemble	0.9742	0.9668	0.9524	0.9785	0.9660	0.9498
	$\alpha = 0.2$	DNN 1	0.9823	0.9677	0.9447	0.9613	0.9634	0.9371
		DNN 2	0.9698	0.9668	0.9403	0.9785	0.9581	0.9485
		DNN 3	0.9760	0.9651	0.9511	0.9613	0.9660	0.9371
		DNN 4	0.9704	0.9625	0.9485	0.9737	0.9634	0.9320
		DNN 5	0.9827	0.9660	0.9460	0.9696	0.9634	0.9377
		Ensemble	0.9827	0.9677	0.9581	0.9767	0.9651	0.9447
	$\alpha = 0.3$	DNN 1	0.9793	0.9660	0.9473	0.9731	0.9677	0.9504
		DNN 2	0.9748	0.9651	0.9422	0.9600	0.9625	0.9396
		DNN 3	0.9767	0.9607	0.9390	0.9763	0.9668	0.9384
		DNN 4	0.9672	0.9677	0.9403	0.9680	0.9642	0.9396
		DNN 5	0.9664	0.9668	0.9365	0.9535	0.9590	0.9473
		Ensemble	0.9787	0.9651	0.9511	0.9728	0.9677	0.9536
	$\alpha = 0.4$	DNN 1	0.9775	0.9625	0.9536	0.9717	0.9642	0.9447
		DNN 2	0.9710	0.9642	0.9504	0.9604	0.9616	0.9447
		DNN 3	0.9829	0.9616	0.9530	0.9769	0.9642	0.9517
		DNN 4	0.9682	0.9651	0.9428	0.9662	0.9442	0.9384
		DNN 5	0.9706	0.9634	0.9479	0.9566	0.9625	0.9390
		Ensemble	0.9789	0.9651	0.9568	0.9729	0.9651	0.9562

Supplementary Table 2: ERM classification accuracy for individual networks and their ensembles for various degrees of mixing (indicated by α) and numbers of epochs. Gray row indicates the ensemble of choice for further analysis.

			Training	Validation	Test
$\alpha = 0$	120 epochs	DNN 1	0.9531	0.9276	0.8825
		DNN 2	0.9420	0.9258	0.8679
		DNN 3	0.9625	0.9267	0.8755
		DNN 4	0.9326	0.9093	0.8545
		DNN 5	0.9423	0.9154	0.8768
		Ensemble	0.9612	0.9346	0.8863
$\alpha = 0.1$	120 epochs	DNN 1	0.9497	0.9285	0.8736
		DNN 2	0.9550	0.9232	0.8774
		DNN 3	0.9788	0.9302	0.8647
		DNN 4	0.9672	0.9258	0.8653
		DNN 5	0.9489	0.9189	0.8710
		Ensemble	0.9703	0.9284	0.8825
$\alpha = 0.2$	120 epochs	DNN 1	0.9537	0.9250	0.8780
		DNN 2	0.9649	0.9197	0.8780
		DNN 3	0.9850	0.9311	0.8780
		DNN 4	0.9398	0.9232	0.8787
		DNN 5	0.9729	0.9302	0.8863
		Ensemble	0.9766	0.9372	0.8907
$\alpha = 0.3$	150 epochs	DNN 1	0.9550	0.9276	0.8831
		DNN 2	0.9676	0.9267	0.8958
		DNN 3	0.9546	0.9302	0.8818
		DNN 4	0.9550	0.9276	0.8818
		DNN 5	0.9734	0.9319	0.8768
		Ensemble	0.9700	0.9389	0.8907
$\alpha = 0.4$	200 epochs	DNN 1	0.9538	0.9328	0.8806
		DNN 2	0.9591	0.9319	0.8723
		DNN 3	0.9620	0.9302	0.8901
		DNN 4	0.9790	0.9433	0.8952
		DNN 5	0.9736	0.9337	0.8768
		Ensemble	0.9746	0.9424	0.8933

A.2 Ablation of ensembling and *mixup*

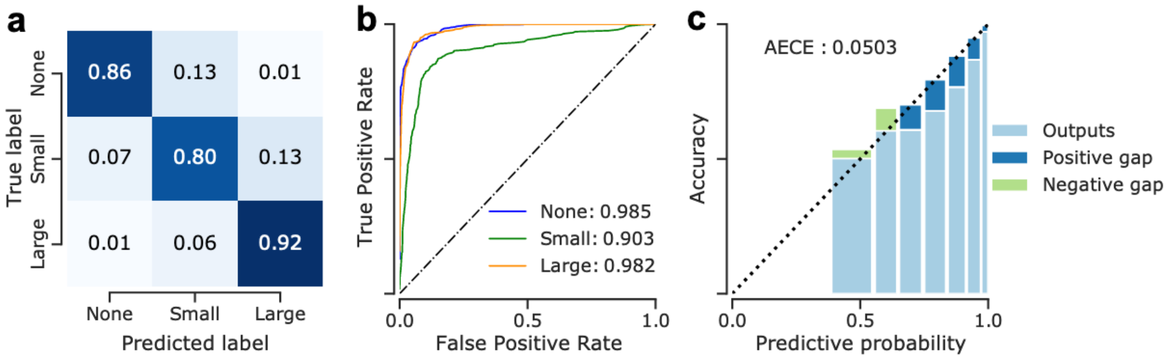

Supplementary Figure 1: Detailed analysis of classification performance of "DNN 1" from the ensemble members trained without *mixup* (Supplementary Table 2) on the test set. **(a)**: Confusion matrix for the model. **(b)**: Receiver operating characteristics of the model. Numbers indicate AUC scores for the respective classes. **(c)**: Reliability diagrams and calibration of the model.
